# Prevalence of depressive disorders in breast cancer patients

**DOI:** 10.1192/j.eurpsy.2024.190

**Published:** 2024-08-27

**Authors:** R. A. Starostin, S. V. Kuzmina, I. G. Gataullin

**Affiliations:** ^1^LLC «Druzhkov Clinic»; ^2^FBEIoHE KSMU MOH Russia; ^3^KSMA - Branch Campus of the FSBEIFPE RMACPE MOH Russia, Kazan, Russian Federation

## Abstract

**Introduction:**

Breast cancer is the most common type of cancer and the leading cause of death from malignant neoplasms in women in Russia and in most countries in the world (Lima *et al*. EClinicalMedicine 2021; 38 100985). According to an analysis of the incidence and mortality from 36 cancers in 185 countries (Sung *et al*. CA Cancer J Clin 2021. 3 209-249) in 2020, 2261419 new cases of breast cancer were identified in the world in both sexes, which is accounted for 11.7% of the total cancer incidence. Mortality from breast cancer in 2020 amounted to 684996 cases. Patients with comorbid depression and anxiety disorders experience more severe symptoms, have longer recovery time, use more healthcare resources and have poorer outcome compare to those with cancer alone (Katon *et al.* Gen Hosp Psychiatry 2007; 2 147-155).

**Objectives:**

Analytical review of data on the impact of depressive spectrum disorders as comorbid conditions on the survival of breast cancer patients and their quality of life.

**Methods:**

The following databases were searched for publications: PubMed, Embase, CINAHL, PsycINFO, Scopus, Science Citation Index/Social Sciences Citation Index, Cochrane Evidence Based Medicine database. The searches were limited to English language and studies with more than 100 subjects with diagnosed breast cancer where this information was mentioned. The analyzed period is between 1977 and 2018.

**Results:**

The reported prevalence of depression in breast cancer patients, according to researches, varies 4,5 to 38%. In patients with I-III stage breast cancer depression increased hazards of all-cause mortality by 50% compared to non-depressed patients. Stage-specific analyses demonstrated a 2–2.5 fold increase in breast cancer-specific and all-cause mortality in patients with stage I and II disease (Vodermaier *et al*. Breast Cancer Res Treat 2014; 2 373-384.). Women with non-metastatic breast cancer who report mild to moderate depressive symptoms in the weeks after surgery have approximately 2.5 times greater risk of death 8–15 years later than women who report little or no depressive symptoms post-surgery (Antoni *et al*. Gen Hosp Psychiatry 2017; 44 16-21). Depression in advanced cancer not only reduces quality of life but is also an independent predictor of poorer survival (Lloyd-Williams *et al.* J Affect Disord 2009; 113 127-132.).

**Conclusions:**

Depression and anxiety both have adverse effects on recurrence and all-cause mortality in patients with breast cancer. Untreated depression leads to significant increase in incidence and mortality. Depression can debut at any stage of cancer, including the stage of diagnosis. It proves the necessity for affective disorders screening in patients with cancer on the stage of diagnosis. Patients with diagnosed affective disorders should be observed not only by oncologist, but also by a psychotherapist in order to receive the necessary treatment to improve the quality of life and reduce the risk of mortality.

**Disclosure of Interest:**

None Declared

